# Autophagy-dependent ferroptosis as a potential treatment for glioblastoma

**DOI:** 10.3389/fonc.2023.1091118

**Published:** 2023-02-10

**Authors:** Yangchun Xie, Tao Hou, Jinyou Liu, Haixia Zhang, Xianling Liu, Rui Kang, Daolin Tang

**Affiliations:** ^1^Department of Oncology, The Second Xiangya Hospital, Central South University, Changsha, China; ^2^Department of Surgery, University of Texas Southwestern Medical Center, Dallas, TX, United States

**Keywords:** autophagy, ferroptosis, glioblasoma, glioblastom stem cells, therapeutics

## Abstract

Glioblastoma (GBM) is the most common malignant primary brain tumor with a poor 5-year survival rate. Autophagy is a conserved intracellular degradation system that plays a dual role in GBM pathogenesis and therapy. On one hand, stress can lead to unlimited autophagy to promote GBM cell death. On the other hand, elevated autophagy promotes the survival of glioblastoma stem cells against chemotherapy and radiation therapy. Ferroptosis is a type of lipid peroxidation-mediated regulated necrosis that initially differs from autophagy and other types of cell death in terms of cell morphology, biochemical characteristics, and the gene regulators involved. However, recent studies have challenged this view and demonstrated that the occurrence of ferroptosis is dependent on autophagy, and that many regulators of ferroptosis are involved in the control of autophagy machinery. Functionally, autophagy-dependent ferroptosis plays a unique role in tumorigenesis and therapeutic sensitivity. This mini-review will focus on the mechanisms and principles of autophagy-dependent ferroptosis and its emerging implications in GBM.

## Introduction

Glioblastoma (GBM) is the most common and aggressive primary brain tumor in adults, with an annual incidence of about 3.23 cases per 100,000 people and a median survival (regardless of treatment) of approximately 8 months, with a one-, five- and ten-year survival rates of 42.8, 7.2 and 4.7%, respectively, based on recent statistical analysis of the Central Brain Tumor Registry of the United States (CBTRUS) (1, 2). Temozolomide is one of the first-line chemotherapeutics for the treatment of GBM due to its DNA alkylating activity and its ability to cross the blood-brain barrier (3). However, GMB patients often develop temozolomide resistance after one year of treatment. One reason for this clinical challenge is that glioblastoma stem cells (GSC) can survive after surgical resection and are highly resistant to chemotherapy and radiotherapy (4). Specifically, autophagy is a cellular recycling mechanism that confers robust chemoresistance and radiation resistance to GSC, resulting in GBM regeneration and the inability to kill them by standard therapies (5). Therefore, understanding the process and function of autophagy is important for developing effective anticancer approaches in GBM.

Autophagy is a catabolic process that promotes the recycling of cellular components under stress conditions (such as nutrient deficiency or microbial infection), thereby restoring cell homeostasis (6). It can be divided into macroautophagy, microautophagy and chaperon-mediated autophagy (CMA) (7). Macroautophagy involves the formation of autophagosomes that encapsulate senescent proteins or damaged organelles into lysosomes for degradation and recycling (8). The macroautophagy process is dynamically mediated by autophagy-related (ATG) family proteins through the formation of distinct protein complexes under the control of post-translational modifications (9). Microautophagy is driven by direct engulfment of cytoplasmic cargo to lysosomes under infectious conditions (10). In CMA, heat-shocked homologous 70 kDa (HSC70) proteins recognize KFERQ motifs in target proteins and facilitate their transfer to lysosomes through the lysosome-associated membrane protein 2A (LAMP2A) receptor (11). This review will focus on macroautophagy, simply referred to as autophagy from now on.

Autophagy is involved in the regulation of various cell death modalities, thereby determining cell fate (12). In addition to promoting cell survival, excessive autophagy can also trigger cell death, especially the iron-dependent form of nonapoptotic ferroptosis. Notably, ferroptosis was originally described as an autophagy-dependent cell death (13). Growing evidence from independent groups highlights that autophagy promotes iron accumulation and lipid peroxidation, key metabolic hallmarks of ferroptosis (14, 15). Consequently, genetic or pharmacological inhibition of the autophagy machinery can suppress ferroptosis sensitivity in various disease models. Moreover, pharmacological induction of autophagy-dependent ferroptosis may be a game-changing antitumor strategy compared to traditional inhibition of autophagy to limit tumor growth (16).

In this review, we summarize the current understanding of the process and basis of autophagy-dependent ferroptosis. We also discuss the implications of induction of autophagy-dependent ferroptosis for the treatment of GBM.

## Molecular mechanism of autophagy-dependent ferroptosis

**Figure 1:**
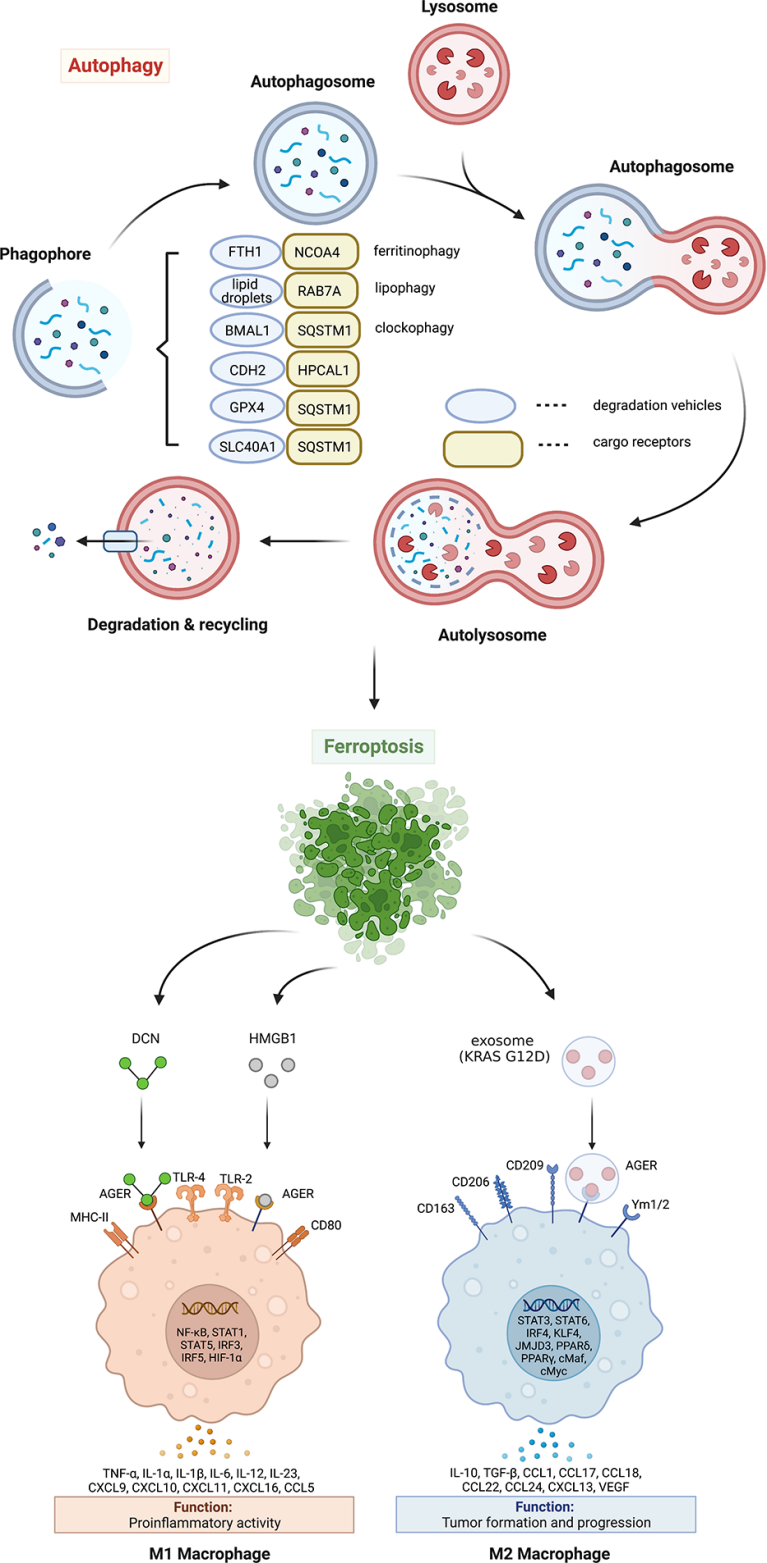
Mechanism and significance of autophagy-dependent ferroptosis. Autophagy promotes ferroptosis by selectively degrading anti-ferroptosis proteins or organelles through multiple autophagy receptors. DAMP release from ferroptotic cells can trigger inflammatory and immune responses in macrophages by activating the AGER pathway. AGER, advanced glycosylation end-product specific receptor; BMAL1, brain and muscle ARNT-like 1; CDH2, cadherin 2; DCN, proteoglycan; FTH1, ferritin heavy chain 1; GPX4, glutathione peroxidase 4; HPCAL1, hippocalcin like 1; HMGB1, high mobility group box 1; IL, interleukin; MHC, major histocompatibility complex; NCOA4, nuclear receptor coactivator 4; RAB7A, member RAS oncogene family; SLC40A1, solute carrier family 40 member 1; SQSTM1, sequestosome 1; TLR, toll-like receptor; TNF, tumor necrosis factor; VEGF, vascular endothelial growth factor.

The activation of autophagy machinery is significantly increased within cells treated with classical ferroptosis inducers, such as small-molecule compounds erastin and RSL3. Compared to wide-type cells, autophagy deficient cells (e.g., ATG5^-/-^ and ATG7^-/-^) exhibit higher survival rate during ferroptosis (17). *In vitro* studies further show that ferroptosis is dependent on autophagy machinery (18, 19). Indeed, excessive formation of autophagosomes or abnormal increase of lysosomal activity will cause the accumulation of intracellular iron and lipid peroxides by selectively degrading proteins regulating iron and redox homeostasis (e.g., ferritin, GPX4, ARNTL, and lipid droplets), promoting the occurrence of ferroptosis (**Figure 1**). The selective role of autophagy in promoting ferroptosis is discussed from the following six aspects.

### Degradation of iron regulatory protein

Excessive ferrous iron can promote the generation of reactive oxygen species (ROS) through Fenton reaction, thus causing toxic effects on cells (20). Under normal physiological conditions, intracellular ferrous iron is absorbed by the ferritin heavy chain 1 (FTH1, also known as FTH). Ferrous iron is then oxidized to ferric iron and stored in ferritin. In addition, excess ferrous iron is transported out of the cell *via* iron exporter solute carrier family 40 member 1 (SLC40A1, also known as ferroportin-1 or FPN1) on the cell membrane (21). Ferroptosis can be induced by increasing iron absorption and decreasing iron storage or preventing iron release. At least two mechanisms mediate iron accumulation and subsequent ferroptosis by promoting autophagic degradation of ferritin or SLC40A1 (**Figure 1**). The degradation of ferritin is mediated by nuclear receptor coactivator 4 (NCOA4)-dependent ferritinophagy in erastin-treated mouse embryonic fibroblasts and human pancreatic cancer cells (17). In contrast, the autophagy receptor sequestosome 1 (SQSTM1, best known as p62) is required for the elimination of SLC40A1 to promote iron-dependent ferroptosis in cancer cells *in vitro* and *in vivo* (22). Although these studies highlight that autophagy increases toxic iron accumulation to induce ferroptosis, whether autophagy selectively affects iron accumulation in different subcellular organelles remains unknown.

### Degradation of lipid droplet

Lipid droplets are highly dynamic organelles that not only store lipids but also release them under stressful conditions. The process of lipid droplet degradation through autophagy is called lipophagy (23). The free fatty acids generated by lipophagy promote adenosine 5′-triphosphate generation through β oxidation in mitochondria. Unlike lipid droplets known to play a role in preventing lipotoxicity by storing fatty acids, lipid droplet degradation mediated by RAB7A lipophagy (**Figure 1**) can promote RSL3-induced lipid peroxidation and ferroptosis in human liver cancer cells (24). In contrast, the overexpression of tumor protein D52 (TPD52) effectively inhibits RSL3-induced lipid peroxidation and ferroptosis by promoting lipid storage or inhibiting lipophagy (24). These findings suggest that lipophagy provides a lipid supply for subsequent lipid peroxidation during ferroptosis. In addition to RAB7A, further identification of lipid droplet-specific autophagy receptors is important for the development of inhibitors targeting lipophagy-dependent ferroptosis.

### Degradation of circadian regulator

The circadian clock is endogenous and controls numerous cellular physiological processes, including iron metabolism, oxidative stress, and cell death, by regulating circadian switches (25). Clockophagy is a type of selective autophagy that degrades circadian rhythm-regulating proteins during ferroptotic cancer cell death (26). The clockophagic degradation of basic helix-loop-helix ARNT like 1 (BMAL1, also known as ARNTL1), the core protein of circadian clock, promotes lipid peroxidation and ferroptosis by increasing lipid storage in droplets through the Egl-9 family hypoxia inducible factor 2 (EGLN2, also known as PHD1)-mediated hypoxia inducible factor 1 subunit alpha (HIF1A) degradation (27). Moreover, SQSTM1 is required for clockophagy-mediated BMAL1 degradation (27) (**Figure 1**), supporting that SQSTM1 is a multisubstrate autophagy receptor for ferroptosis.

### Degradation of GPX4

Glutathione peroxidase 4 (GPX4), formerly known as phospholipid hydrogen peroxide glutathione peroxidase (PHGPx), is one of the core regulators and targets of ferroptosis (28). GPX4 is the fourth member of the selenium-containing GPX family with a unique ability to scavenge membrane lipid hydroperoxide products to alcohols (29). In 2014, a targeted metabolomics study showed that the overexpression or knockdown of GPX4 can regulate the cytotoxicity of 12 ferroptosis inducers (28). Mechanistically, GPX4 uses its catalytic activity to weaken lipid peroxide toxicity and maintain membrane lipid bilayer homeostasis. RSL3, an inhibitor of GPX4, covalently binds to GPX4 and inactivates GPX4, leading to the accumulation of intracellular peroxides and triggering ferroptosis (28). As a cofactor of GPX4, glutathione (GSH) deficiency inactivates GPX4 and triggers ferroptosis. Therefore, the inhibition of GPX4 activity and the decrease of GPX4 expression can destroy the balance of cellular redox system, causing the accumulation of lipid ROS and ferroptosis. Both erastin and RSL3 induce autophagy flux and affect GPX4 levels through SQSTM1-mediated GPX4 protein degradation in multiple cancer cells (22) (**Figure 1**). Moreover, pharmacological inhibition of mammalian target of rapamycin complex 1 (mTORC1) by rapamycin also reduces GPX4 protein levels, while *vice versa* RSL3 inhibits mTORC1, supporting a relationship between autophagy and ferroptosis (30). FIN56, another ferroptosis inducer, also promotes GPX4 protein degradation and lipid peroxidation in an autophagy-dependent manner (31). Since CMA also mediates ferroptosis machinery protein degradation, such as GPX4 and acyl-CoA synthetase long-chain family member 4 (ACSL4) (32, 33), the receptors of which are heat shock proteins, it is necessary to further elucidate the roles of different types of autophagy in promoting ferroptosis.

### Degradation of CDH2

Historically, hippocalcin like 1 (HPCAL1) is a neuron-specific Ca2+-binding protein that control central nervous system responses (34). In terms of tumor formation and development, HPCAL1 exhibits tumor-promoting activity in GBM by the activation of the embryonic developmental signals, especially the WNT-CTNNB1/β-catenin pathway (35). Recently, HPCAL1 was identified by quantitative proteomic approach as a novel autophagy receptor that triggers autophagy-dependent ferroptosis by selectively degrading cadherin 2 (CDH2) (**Figure 1**) (36). Mechanistically, the degradation of CDH2 is initiated by protein kinase C theta (PRKCQ)-mediated HPCAL1 phosphorylation on Ter149. Notably, starvation-induced autophagy does not require HPCAL1, which establishes the first autophagy receptor to induce ferroptosis. Furthermore, transmembrane protein 164 (TMEM164) acts as a specific promoter of ferroptosis-related autophagosome formation, but not ATG9A-dependent and starvation-induced autophagosome formation (37). These studies provide important insights into the upstream signaling and downstream mediators of autophagy-dependent ferroptosis.

### Organelle-specific initiation of autophagy-dependent ferroptosis

**Figure 2:**
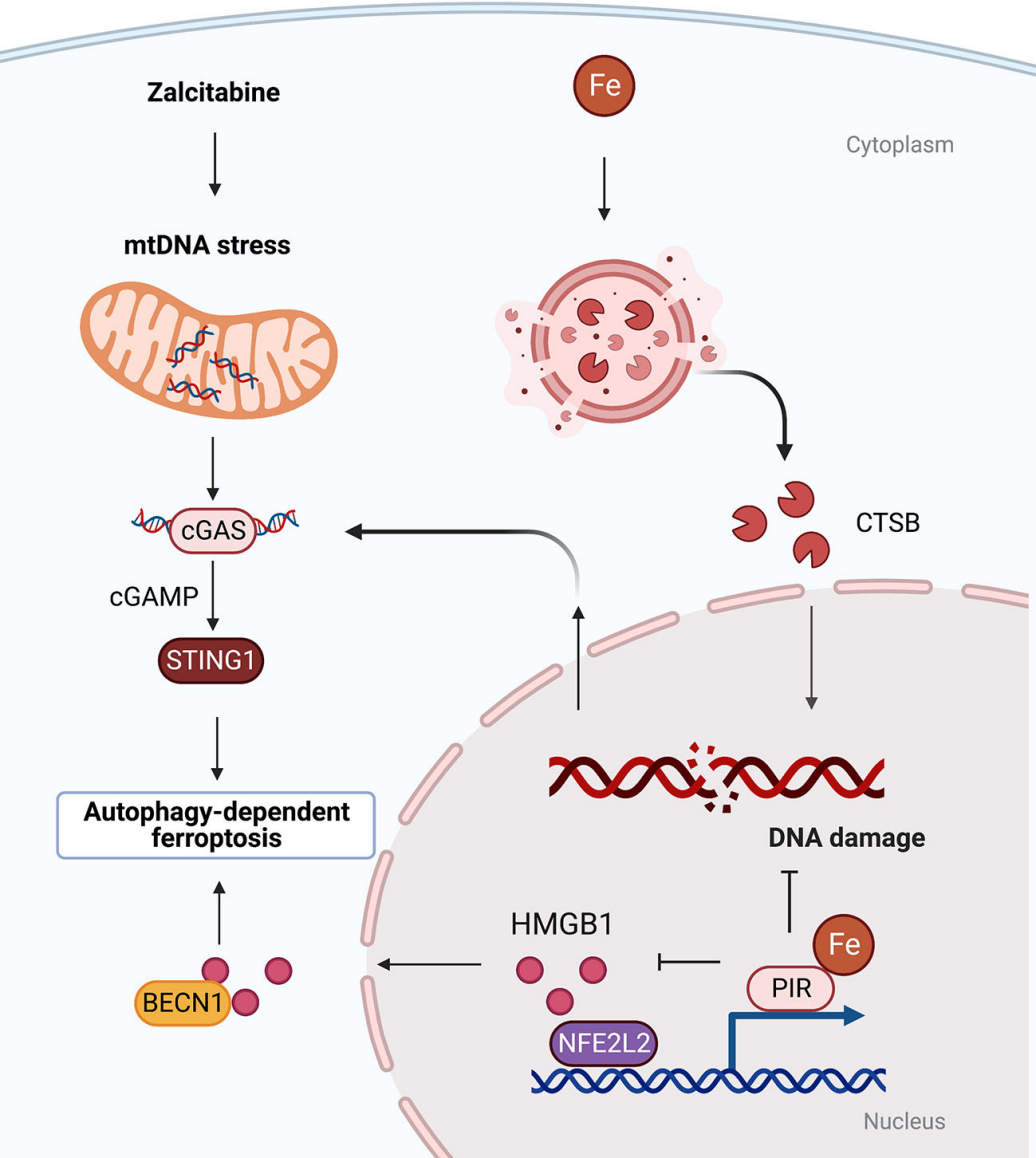
Organelle-specific initiation of autophagy-dependent ferroptosis. There is crosstalk between lysosomes, nucleus and mitochondria. Oxidative damage to nuclear or mitochondrial DNA triggered by iron-dependent CTSB translocation or anti-HIV drug zalcitabine can activate STING-dependent autophagy and ferroptosis. BECN1, beclin 1; cGAS, cyclic GMP-AMP synthase; CTSB, cathepsin B; HMGB1, high mobility group box 1; PIR, pirin; STING1, stimulator of interferon response cGAMP interactor 1.

In addition to lipid droplets, other organelles play context-dependent roles in mediating autophagy-dependent ferroptosis (38). For example, the lysosomal cysteine protease cathepsin B (CTSB) promotes autophagy-dependent ferroptosis *via* translocation from lysosome into nucleus to cause DNA damage signals and to activate stimulator of interferon response cGAMP interactor 1 (STING1, also known as STING or TMEM173)-dependent DNA sensor pathways (39) (**Figure 2**). In addition, the MAPK-STAT3-CTSB pathway is required for erastin-induced ferroptosis in pancreatic cancer cells (40). The inhibition of STAT3 through small molecules (e.g., cryptotanshinone and S31-201) or siRNA as well as blockade of CTSB activity (using CA-074Me) or vacuolar type H+-ATPase (using bafilomycin A1) limits ferroptosis (40). These findings suggest that there is organelle communication between the lysosome and the nucleus to initiate autophagy-dependent ferroptosis.

Mitochondria play an important role in the process of ferroptosis, including participating in mitochondrial DNA biosynthesis, ROS metabolism and mitochondrial iron storage and transport (41, 42). The DNA sensor hub STING1 links mitochondrial DNA damage, autophagy, and ferroptosis. Specifically, anti-HIV drug zalcitabine-induced mtDNA depletion and oxidative DNA damage activates the cGAS-STING1 pathway, thereby triggering STING1-dependent autophagy and subsequent autophagy-mediated ferroptosis (43) (**Figure 2**). Mitochondria associating with lipid droplets in fat-oxidizing tissues are recently identified as peidroplets mitochondria, which have unique ATP synthesis and pyruvate oxidation capacities (44), potentially suggesting a functional role in autophagy-dependent ferroptosis through lipophagy. Furthermore, the iron-binding nuclear protein pirin (PIR) can hijack HMGB1 in the nucleus, thereby inhibiting the translocation of HMGB1 to the cytoplasm and subsequent activation of beclin 1 (BECN1)-dependent autophagy and ferroptosis in pancreatic cancer cells (45, 46). These findings explain the persistent activation of DNA damage, DAMP release, and autophagy flux during ferroptotic death.

## Targeting autophagy-dependent ferroptosis signaling network in GBM

GBM cells promote autophagy under adverse conditions (e.g., nutrient deficiency, oxidative or hypoxic stress) to maintain their survival and evade responses to cancer therapy (47–49). The progression of GBM is associated with decreased autophagy capacity (50). In a *KRAS*-driven mouse model of GBM, inhibition of ATG significantly reduced tumor growth and oncogenic progression, suggesting that autophagy is critical for GBM initiation and growth (51). While temozolomide induces autophagy to kill GBM, GSCs exert self-protection by activating autophagy (52). The current study aims to find new therapeutic targets to improve patient outcomes. Recent studies have shown that ferroptosis exists in GBM tumor cells, and recurrent tumors are more prone to ferroptosis treatment (53). These results confirm that exploiting the ferroptosis process may be a possible new therapeutic strategy, especially in the setting of recurrent GBM.

There is also evidence that GBMs have significantly increased iron requirements compared to normal tissues, and GSCs uptake twice as much iron as non-stem tumor cells (54). Thus, GBM cells have a strong iron reliance. Targeting iron-related proteins or increasing intracellular iron levels are considered as feasible methods for GBM treatment (**Table 1**). Amentoflavone induces ferroptosis in glioma cells though ATG7-mediated autophagy to break iron homeostasis (55). Interestingly, both coatomer protein complex subunit zeta 1 (COPZ1) and tripartite motif containing 7 (TRIM7) are associated with ferroptosis by regulating intracellular iron metabolism in GBM (57, 59). Genetic inhibition of COPZ1 or TRIM7 suppresses tumor growth *in vitro* and *in vivo*, mechanistically by inducing NCOA4 expression and promoting ferritinophagy, followed by increased intracellular levels of ferrous iron and ultimately ferroptosis (57, 59). More recently, multifunctional nanomaterials (including ultrasmall iron oxide nanoparticles and iron oxide nanoparticles loaded with paclitaxel) have the effects of increasing the intracellular iron level, catalyzing fenton reaction, generating ROS and lipid peroxidation, ultimately inducing ferroptosis *via* a BECN1-dependent autophagy pathway (56, 58).

**Table 1:** *In vivo* or *in vitro* studies targeting autophagy-dependent ferroptosis in GBM.

Compounds/methods	Targets of autophagy-dependent ferroptosis	*In vitro* models	*In vivo* models	Refs.
Amentoflavone	ATG7-dependent autophagy	Human GBM cell lines U251 and U373	BALB/c nude mice bearing subcutaneous xenograft	(55)
Ultrasmall iron oxide nanoparticles (USIONPs)	Beclin1/ATG5-dependent autophagy	Human GBM cell line U251	NA.	(56)
Silencing COPZ1	NCOA4 mediated autophagy	Human GBM cell line U87MG and U251	Nude mice bearing intracranial xenograft tumors	(57)
Iron oxide nanoparticles loaded with paclitaxel (IONP@PTX)	Degradation of GPX4	Human GBM cell line U251	BALB/c-nu mice bearing GBM xenografts	(58)
Silencing TRIM7	NCOA4-mediated ferritinophagy	Human GBM cell line A172 and U87MG	Non-obese diabetic-severe combined immunodeficient NOD-SCID mice bearing subcutaneous and intracranial tumor xenograft	(59)

In addition to iron addiction, GBM has a strong capacity of lipid synthesis, which is related to its malignant degree. Breaking lipid metabolism balance in GBM can induce ferroptosis to inhibit tumor growth. Therefore, exploiting this metabolic alteration in GBM to induce ferroptosis may be another effective therapeutic direction.

The damage-associated molecular patterns (DAMPs) released by dead, dying, or stressed cells act as alarm signals to trigger innate and adaptive immune responses (60). The early release of high mobility group box 1 (HMGB1), proteoglycan core proteoglycan (DCN), or mutated KRAS-G12D protein during ferroptosis is an active process involving secretory autophagy, lysosomal exocytosis, and exosome secretion (61–63) (**Figures 1**, **2**). Once released by ferroptotic cells, these extracellular DAMPs bind to the receptor advanced glycosylation end-product specific receptor (AGER) on macrophages and trigger either proinflammatory cytokine production in a nuclear factor-κB (NF-κB)-dependent manner or macrophage polarization-associated tumor progression (**Figure1**). Hypoxic glioma-derived exosomes promote M2-like macrophage polarization by enhancing autophagy induction (64). Taken together, pharmacological or genetic inhibition of the DAMP-AGER axis can limit the ability of ferroptotic cancer cells to induce tumor-protective immune responses.

## Conclusion and outlook

Autophagy is a degradation process controlled by a cascade of ATG protein complexes, each of which regulates different stages of initiation and formation of autophagic membrane structures. Compared with autophagy to promote cell survival, the molecular mechanism by which autophagy promotes cell death is poorly understood (65, 66). The discovery of ferroptosis as an autophagy-dependent cell death provides an opportunity to suppress cancers with excessive autophagy (67). Selective autophagic degradation of anti-ferroptosis proteins or organelles promotes iron-dependent oxidative damage and cell death. For GBMs, induction of autophagy-dependent ferroptosis facilitates clearance of drug-resistant cancer stem cells. Although several experimental ferroptosis activators are available, there is still a shortage of related drugs that can be used in clinical trials. In the future, we need more in-depth work to determine the specific mechanism of autophagy-dependent ferroptosis (68), identify circulating biomarkers to monitor the activity of this pathway (69), and design the next generation of ferroptosis-related drugs (70).

## Author contributions

YX proposed the research. YX and TH both reviewed the literature and collected references. YX, RK, and DT wrote the manuscript and finalized the paper. JL provided some of the material. HZ and XL have adjusted some expressions of the article. All authors contributed to the article and approved the submitted version.
